# An ultra-sensitive and rapid immunosensor for the onsite detection of circulating tumor DNA in breast cancer

**DOI:** 10.3389/fbioe.2024.1412598

**Published:** 2024-07-12

**Authors:** Yi Bi, Xiao Lv, Ke Wang, Jinyu Wu, Xiang Shi, Xiaodong Zheng, Xiaogang Lin

**Affiliations:** ^1^ Chongqing University Cancer Hospital, Chongqing University, Chongqing, China; ^2^ Key Laboratory of Optoelectronic Technology and Systems of Ministry of Education of China, Chongqing University, Chongqing, China; ^3^ Chongqing Key Laboratory of Translational Research for Cancer Metastasis and Individualized Treatment, Chongqing, China; ^4^ Affiliated Hospital of Chongqing Medical and Pharmaceutical College, Chenjiaqiao Hospital of Shapingba District, Chongqing, China

**Keywords:** affinity sensor, capacitive biosensor, circulating tumor DNA, PIK3CA-H1047R, alternating current electrokinetics

## Abstract

Breast cancer currently stands as the most prevalent form of cancer worldwide and the primary cause of cancer-related deaths among women. However, the current diagnostic methods for breast cancer exhibit several limitations, including invasiveness, high costs, and limited sensitivity and specificity. The detection of the PIK3CA-H1047R variant is of paramount importance due to its close association with tumor growth and treatment resistance. Consequently, developing a straightforward, rapid, and highly sensitive approach for detecting PIK3CA-H1047R is of utmost importance. We have been working on the development of a rapid and ultrasensitive biosensor, leveraging the alternating current (AC) electrokinetic (ACEK) capacitive sensing method. This biosensor involves modifying the surface of interdigital electrodes with antibodies, facilitating the antibody–antigen-binding process through AC electrokinetic techniques. Our sensor strategy directly measures the interface capacitance, and the rate of change serves as a quantitative marker for event identification. Remarkably, our biosensor successfully detects the PIK3CA-H1047R antigen within a concentration range of 1 ng/mL to 1 μg/mL. In conclusion, this study proposes a fast and highly sensitive biosensor for the detection of a key breast cancer marker, the PIK3CA-H1047R variant. This technology is expected to improve breast cancer diagnosis, address the limitations of current methods, and provide patients with better treatment options. This detection method offers a promising avenue for on-site and real-time sensitive detection of the PIK3CA-H1047R antigen, potentially revolutionizing breast cancer diagnosis.

## 1 Introduction

Breast cancer is one of the most harmful cancers among women, accounting for 24.2%, and has the highest cancer incidence (Sung et al., 2021). More than 2.3 million people are diagnosed with breast cancer worldwide, causing more than 650,000 estimated deaths per year (Siegel et al., 2020; Sung et al., 2021; Siegel et al., 2022). Studies have found that early screening for breast cancer significantly reduces mortality, and countries with lower breast cancer mortality rates are characterized by increased levels of coverage of essential health services. Some of these countries have population-wide breast cancer screening programs (Siu and on behalf of the U.S. Preventive Services Task Force, 2016; Duggan et al., 2021; Sung et al., 2021). Screening for breast cancer includes mammography, clinical breast screening (CBE), digital mammography (DBT), breast ultrasound (BUS), and magnetic resonance imaging (MRI) (Surendra et al., 2021). Although mammography is the only screening test that has been demonstrated to reduce the death rate by at least 20 percent (Uematsu, 2017), it has high false-positive diagnoses. Furthermore, currently, the selection of breast cancer treatment is based on the analysis of tumor biopsy, but changes could occur during cancer treatment, which makes treatment options more experience-dependent (Sant et al., 2022).

With the advancement of detection technology, studies have found that liquid biopsy can screen for early tumors and monitor tumor changes (The TRACERx consortium, The PEACE consortium, 2017; Castelli et al., 2018). Circulating tumor DNA (ctDNA)-based biomarkers have also been widely investigated. At present, many gene loci in ctDNA are diagnostic markers for breast cancer, such as *PIK3CA*, *BRCA2*, *NBNPTEN*, *ESR1*, *AKT1*, *HER2*, *TP53*, and *GATA3* genes (Walsh et al., 2006; Berns et al., 2007; Jones et al., 2010; Janku et al., 2012; Li et al., 2013; Hosoda et al., 2014; Jeselsohn et al., 2014; Kuchenbaecker et al., 2017). The mutation of the *PIK3CA* gene encoded by phosphatidylinositol3-kinase, PI3K, is the most likely mutated gene in breast cancer mutated genes except *HER2* and *TP53*. About 90% of PIK3CA mutations occurred at H1047R, E545K, and E542K, while mutations at H1047R accounted for about 50% (Yuan et al., 2019).

Methods currently used to detect PIK3CA mutations in breast cancer patients include Sanger sequencing, PCR-RFLP, next-generation sequencing (NGS), and digital PCR (dPCR). Sanger sequencing is a common DNA sequencing method for known mutations in the *PIK3CA* gene (Arsenic et al., 2015). PCR-RFLP amplifies specific *PIK3CA* gene regions via PCR and detects mutations through enzyme digestion (Li et al., 2016). NGS is a high-throughput sequencing technology for simultaneous mutation detection in multiple genes, providing comprehensive analysis (Lee et al., 2024). Digital PCR is highly sensitive, enabling absolute quantification of mutations, and is especially useful for detecting very low-frequency mutations (Oshiro et al., 2015). However, these detection methods require complicated operation, expensive equipment, professionals, high detection costs, and long detection times.

Herein, to solve the present problem of detecting the PIK3CA-H1047R antigen, we develop a novel PIK3CA-H1047R biosensor based on the alternating current (AC) electrokinetics (ACEK) effect. First of all, the PIK3CA-H1047R antibody was modified on the surface of the interdigital electrode using a self-assembly technique (Parviz et al., 2003; Ye et al., 2017), which was used as the specific capture probe of the PIK3CA-H1047R antigen. The PIK3CA-H1047R antigen was enriched by an AC electrokinetics effect generated by the symmetrical electrode of the interdigital electrode. The specific binding of the antibody and antigen changes the interfacial capacitance of the electrode surface, so the trace measurement of the PIK3CA-H1047R antigen can be realized through the relative change of interfacial capacitance. Compared with the traditional ctDNA detection method, this method can detect a trace of 10 uL within 1 min and is simple to operate without labeling the test object to be measured, providing a development direction for mobile medical testing. Through this sensor, we can rapidly and accurately detect breast cancer-related antigens, thereby enabling earlier detection and diagnosis of breast cancer and providing patients with more timely treatment and intervention. Additionally, the ease of operation and rapid detection capabilities of this technology hold the promise of offering breast cancer patients more convenient and widespread diagnostic and therapeutic services, thereby providing a new direction and potential for the development of the mobile healthcare field.

## 2 Sensing and enrichment mechanisms

**FIGURE 1:**
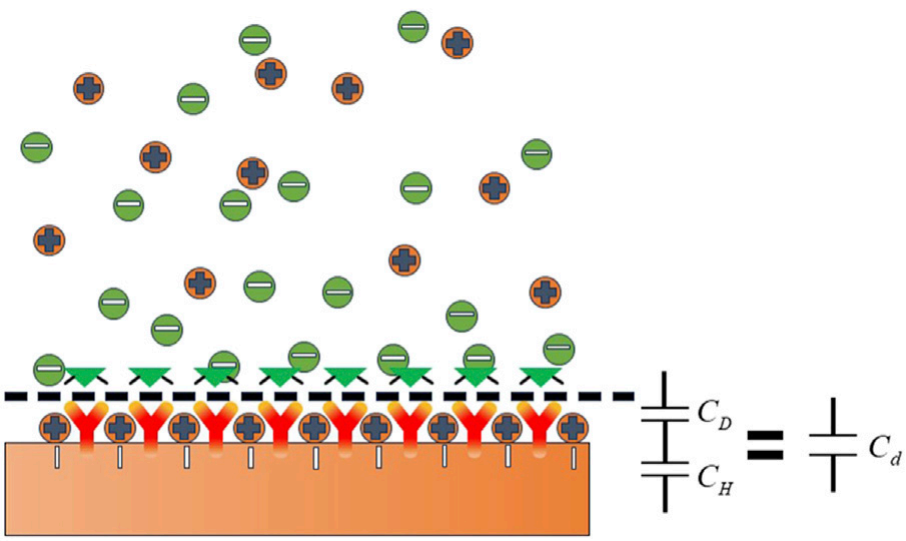
Double-layer schematic diagram.

When the electrode comes into contact with a liquid electrolyte, some charged particles in the solution or dipole electrics selectively enrich the surface, forming the double electric layer near the electrode interface, as shown in Figure 1 (Grahame, 1947).

The capacitance of the double electric layer can be expressed by Eq. 1

$$\frac{1}{C_{d}}=\frac{1}{C_{H}}+\frac{1}{C_{D}}.$$ (1)

**FIGURE 2:**
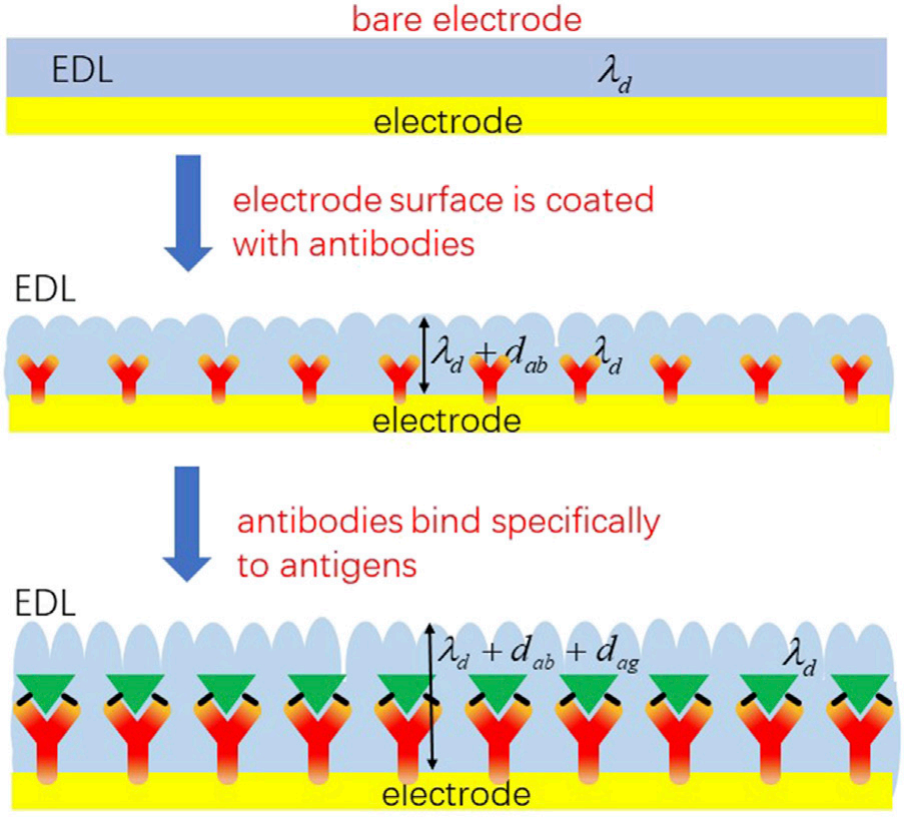
Schematic diagram of the electrode interface capacitance change.

The charging and discharging processes of the double electric layer are similar to those of parallel plate capacitance, so it can be considered equivalent to parallel plate capacitance. As shown in Figure 2, when the biomolecular solution is in contact with the double electric layer, the electrode surface area $A_{0}$ without any modification, the capacitance of the double electric layer $C_{\mathrm{int},0}$ can be expressed by Eq. 2

$$C_{\mathrm{int},0}=\frac{\varepsilon_{A}A_{0}}{\lambda_{d}}.$$ (2)

When the antibody with a thickness of $d_{ab}$ is fixed to the electrode surface through a self-assembly, the thickness of the double electric layer becomes $\lambda_{d}+d_{ab}$ , the surface area becomes $A_{ab}$ , and the capacitance of the electric double layer $C_{\mathrm{int},ab}$ can be expressed by Eq. 3

$$C_{\mathrm{int},ab}=A_{ab}/\left(\frac{\lambda_{d}}{\varepsilon_{A}}+\frac{d_{ab}}{\varepsilon_{ab}}\right),$$ (3)

where $\varepsilon_{A}$ is the permittivity of the solution and $\varepsilon_{ab}$ is the permittivity of the antibody. After the antibody is stably fixed to the electrode surface, the antigen solution is dropped onto the electrode surface to specifically combine with the antibody, and the interfacial capacitance change can be expressed by Eq. 4

$$C_{\mathrm{int},a,g}=A_{ag}/\left(\frac{\lambda_{d}}{\varepsilon_{A}}+\frac{d_{ab}}{\varepsilon_{ab}}+\frac{d_{ag}}{\varepsilon_{ag}}\right),$$ (4)

where $A_{ag}$ is the interface capacitance surface area after antibody–antigen binding, $d_{ag}$ is the thickness of the antigen, and $\varepsilon_{ag}$ is the permittivity of the antigen.

In this work, binding between the PIK3CA-H1047R antigen and PIK3CA-H1047R antibody was marked with changing $C_{\mathrm{int}}$ . The normalized capacitance change rate is used to reflect the binding speed. The normalized change in capacitance is represented by Eq. 5

$$\frac{\Delta C}{C_{\mathrm{int},ab}}=-d_{ag}/\left(\frac{\lambda_{d}}{\varepsilon_{A}\varepsilon_{ag}}+\frac{d_{ab}}{\varepsilon_{ab}\varepsilon_{ag}}\right).$$ (5)

Therefore, $\Delta C/C_{\mathrm{int},ab}$ can be directly correlated with the number of bounded micromolecules on the electrode surface, thus enabling quantitative detection of the target to be measured. In addition, the use of the normalized capacitance change rate $\Delta C/C_{\mathrm{int}}$ helps improve the repeatability of the test, and it is independent of the actual operating area of the sensor.

ACEK effects are a group of microflow phenomena under the action of nonuniform alternating electric fields. They can be used to induce directed movement of microflows and particles in the solution so that the analytes in the solution can be quickly routed toward the surface of biosensors, thus greatly improving the binding efficiency. ACEK effects mainly include the dielectrophoretic (DEP) effect, alternating current electroosmosis (ACEO) effect (Wu et al., 2005; Hart et al., 2010), and alternating current electrothermal (ACET) effect (Lian et al., 2007). The DEP effect induces the movement of particles toward the sensor surface, while the ACEO and ACET effects can produce microflows that accelerate the binding of the object under test.

In our work, without an AC signal, antigens move randomly and take hours or even days to get the result. However, using the AC signal, the ACEK effect caused microflow and particles’ directional movement, accelerated antigen binding on the surface of the electrode, and the detection time is expected to be reduced to 60 s.

## 3 Materials and methods

### 3.1 Experimental equipment

The experimental equipment mainly includes a constant temperature chamber (FYL-YS-100L, Beijing Fuyi Electric Co., Ltd.), a CNC ultrasonic cleaner (KQ-300DE, Kunshan Ultrasonic Instrument Co., Ltd.), an ultrapure water machine (Molresearch 1006 a, Chongqing Molresearch Water Treatment Equipment Co., Ltd.), an ultra-low temperature freezer (DW-HL100, low-temperature technology, Zhongke Meiling Cryogenics Co., Ltd.), a UV ozone cleaner (SDP-UVT, NovaScan Inc., United States), an impedance analyzer (IM3536, measurement technology, Hioki (Shanghai) Co., Ltd.), and an XPS instrument (K-Alpha+, Thermo Fisher Scientific, United States).

### 3.2 Chemical reagents

Chemical reagents included 1 × PBS buffer (purchased from Beijing Solebo Technology Co., Ltd.), 11-mercaptoundecanoic acid (MUA, purchased from Shanghai Yuanye Bio-Technology Co., Ltd.), N-hydroxysuccinimide (NHS, 98%), 1-(3-dimethylaminopropyl)-3-ethylcarbodiimide hydrochloride (EDC, 98.5%, purchased from Sigma Corporation, United States), acetone (purchased from Chongqing Chuandong Sichuan Engineering Co., Ltd.), ethanolamine, isopropyl alcohol, and anhydrous ethanol (purchased from Shanghai MacLean Biochemical Technology Co., LTD.). The PIK3CA-H1047R antibody and PIK3CA-H1047R antigen were all purchased from Wuhan Biotechnology Co., Ltd. The serum was ordered from Chongqing University Cancer Hospital.

The MUA solution with a concentration of 5 mmol/L was obtained by dissolving 11-mercaptoundecanoic acid in anhydrous ethanol, and 0.4 mol/L of the EDC solution was prepared by dissolving EDC in a 1 × PBS buffer solution. NHS was dissolved in a 1 × PBS buffer solution to prepare a 0.1 mol/L NHS solution. Then, 0.4 mol/L of the EDC solution is mixed with 0.1 mol/L of the NHS solution in a volume ratio of 1:4 to obtain the EDC/NHS activation solution. Ethanolamine was dissolved in a 1 × PBS buffer solution to prepare 1 mol/L ethanolamine blocking solution.

The concentration of the 0.1 g/mL PIK3CA-H1047R antibody was diluted into two antibody concentrations of 1 μg/mL and 1 ng/mL. The concentration of 1 μg/mL PIK3CA-H1047R antigen was diluted into four concentrations of 1 μg/mL, 100 ng/mL, 10 ng/mL, and 1 ng/mL solutions. To detect the matrix effect of serum, 10 μg/mL of the antigen solution was added to the human serum and diluted with a 1 × PBS buffer solution. The final concentrations of antigen in serum samples were 1 μg/mL, 100 ng/mL, 10 ng/mL, and 1 ng/mL, respectively.

### 3.3 Preparation of microelectrode sensor chips

**FIGURE 3:**
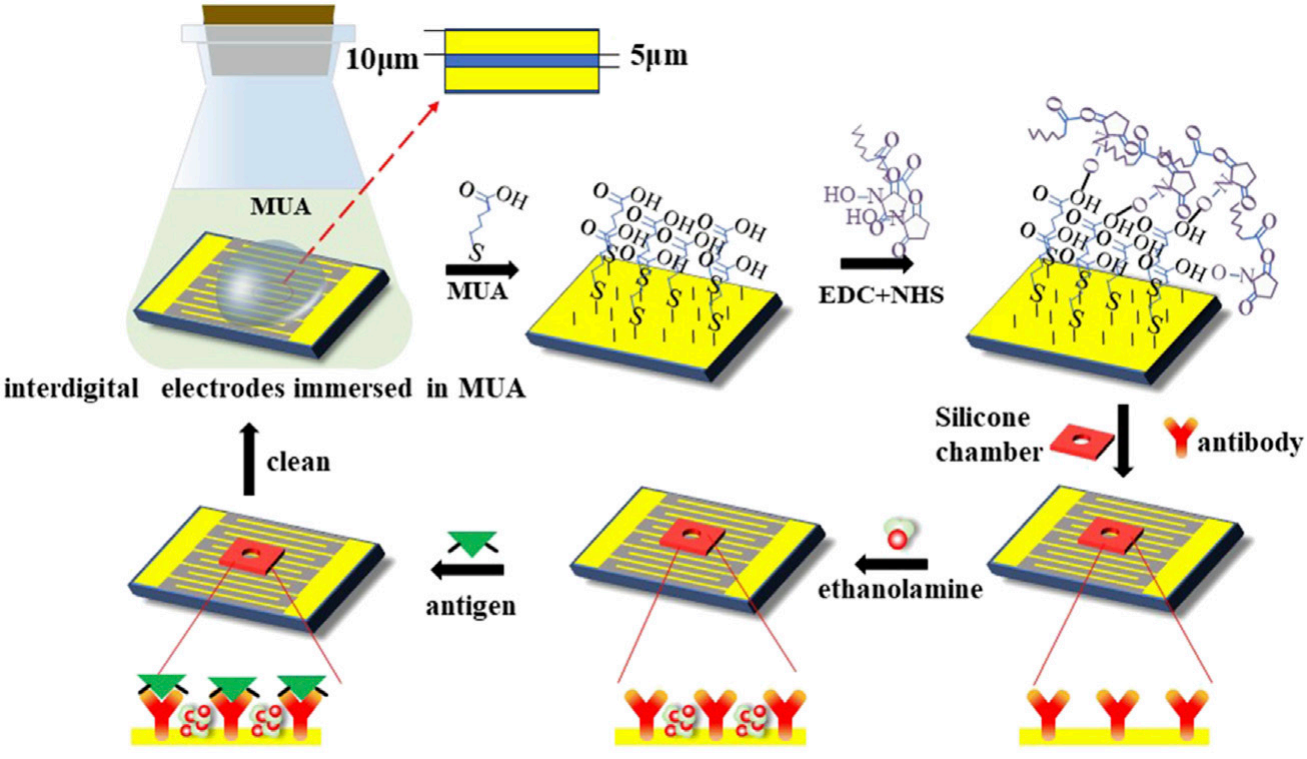
Representation of surface modification techniques on the interdigital electrodes’ surface for the detection of the PIK3CA-H1047R antigen.

In this study, the interdigital electrodes were employed as a sensing chip for ACEK-binding acceleration and impedance measurements of serum samples. Gold-planar microelectrodes were fabricated on silicon wafers. The microelectrode arrays had a feature length of 10 μm (10 μm width and 5 μm spacing). Before detection, the interdigital electrode surface needs to be functionalized. The specific steps are shown in Figure 3.

Before detection, the interdigital microelectrodes should be modified with the following steps sequentially: they were soaked in acetone for 5 min with ultrasonic cleaning; rinsed in isopropyl alcohol (IPA) and deionized water for 30 s, respectively; and dried with an air gun. Cleaned chips were transferred into a UV ozone cleaner and treated for 15 min to increase the hydrophilicity of the electrode surface. Then, the chips were soaked in the 5 mmol/L MUA solution and placed in the incubator for 12 h at a temperature of 25°C, which can form the Au–S bonds and realize the self-assembled monolayer. Third, the EDC and NHS solutions were added to the electrodes, and the electrode’s surface should be cleaned with absolute ethyl alcohol and blow-dried with nitrogen; then, the sensors should be placed in an incubator for 2 h. After the activation of the carboxyl group, the electrodes’ surface should be cleaned with deionized water and blow-dried with nitrogen; then, the chambers should be pasted on the sensors. After that, the PIK3CA-H1047R antibody was immobilized onto the electrode surface in a humidor for 3 h at 37°C. Finally, the electrodes were blocked with the ethanolamine blocking solution at a concentration of 1 mol/L for 1 h at 25°C to inhibit non-specific binding.

### 3.4 Measurement procedure

A measure of 10 μL of the sample was dropped onto the functionalized sensor, which was connected to a high-precision impedance analyzer (Agilent, 4294A), and the capacitance was then measured under a 300-mV RMS AC voltage at 20 kHz for 60 s. Then, the rate of change of normalized capacitance was calculated to represent the binding level of antigen, and the slope of normalized capacitance with time (%/min) was obtained by the least-squares linear fitting method. The normalized capacitance is calculated as C_t_/C_0_, where C_t_ and C_0_ are the capacitance values at time t and zero, respectively.

## 4 Results and discussions

### 4.1 Detection of the PIK3CA-H1047R antigen in analytical samples

To evaluate the performance of this method, four concentrations of 1 μg/mL, 100 ng/mL, 10 ng/mL, and 1 ng/mL diluted antigen solution in 1 × PBS were tested. The sensor capacitance was measured with an AC signal of 300 mV RMS at 20 kHz for 60 s. During the test, the capacitance changed linearly with time, and with the increase in the concentration of the PIK3CA-H1047R antigen, that is, the increase in the binding level of the PIK3CA-H1047R antigen and antibody, the change in capacitance also increased. The slope of these capacitance curves was obtained by least-squares linear fitting as a quantitative indication of the binding rate of the antigen and antibody on the electrode surface. The change rates were found to be −1.306%/min, −1.659%/min, −2.548%/min, and −3.275%/min for PIK3CA-H1047R antigen levels at 1.0 ng/mL, 10 ng/mL, 100 ng/mL, and 1 ug/mL, respectively.

**FIGURE 4:**
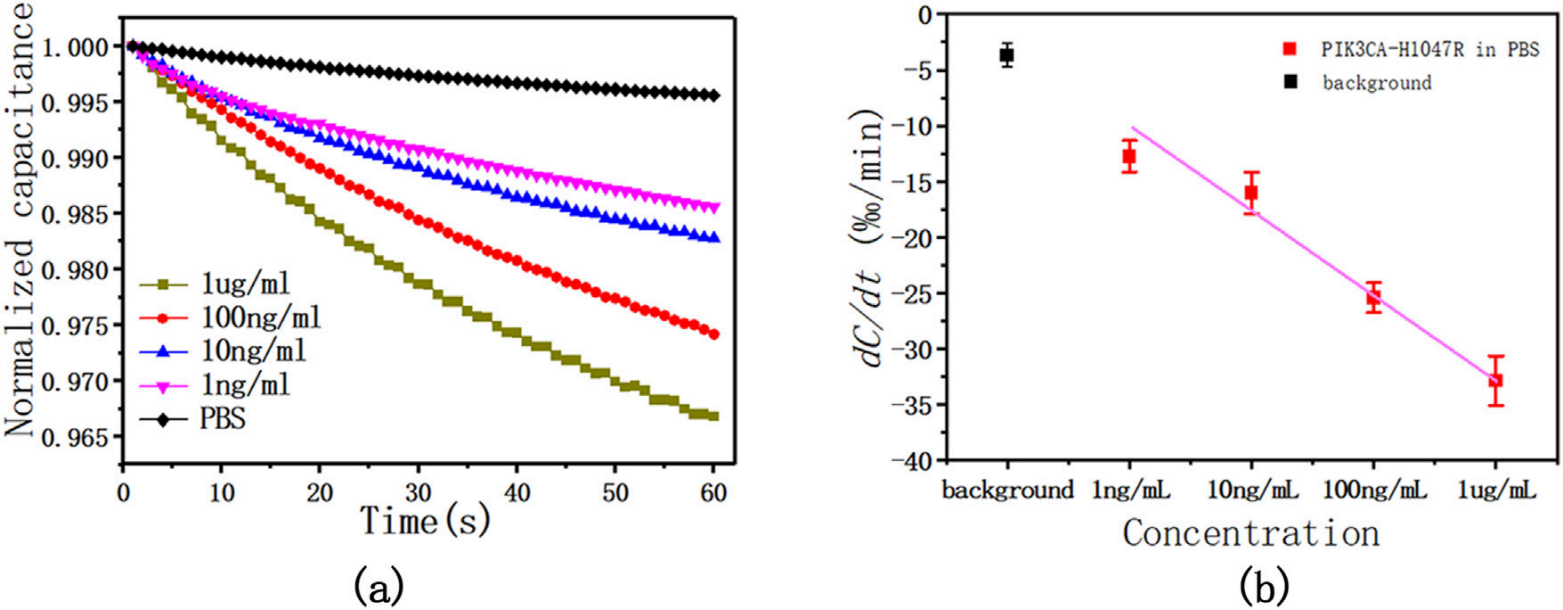
**(A)** This is a representative curve of normalized capacitance at different PIK3CA-H1047R antigen concentrations, showing the dC/dt curve as a function of time within 60 s at various levels of the PIK3CA-H1047R antigen spiked in 1 x PBS. **(B)** Capacitance change rate as a function of PIK3CA-H1047R antigen concentrations in 1 × PBS. The AC signal used was 300 mV RMS at 20 kHz.

The tests shown in Figure 4A were performed five times. Figure 4B shows the averages and standard deviations (SDs) of the sensor responses, which also shows a correlation between PIK3CA-H1047R antigen concentrations and capacitance change rates. The PIK3CA-H1047R antigen samples showed change rates of −1.274% ± 0.141%/min, −1.602% ± 0.188%/min, −2.543% ± 0.134%/min, and −3.285% ± 0.223%/min for 1.0 ng/mL to 1 ug/mL of the PIK3CA-H1047R antigen, respectively (Figure 2B). Within the range of 1.0 ng/mL to 1 ug/mL PIK3CA-H1047R antigen, dC/dt exhibited a logarithmic dependence on the PIK3CA-H1047R antigen concentration. A linear, inverse association between dC/dt and PIK3CA-H1047R antigen concentration was observed. The dependence is expressed as

$$Y\left(\%/\min\right)=-7.106\log\left(\text{PIK}3\text{CA}-H1047R\text{ antigen concentrations in }1\times \text{PBS}\right)+3.073.$$ (6)

Pearson correlation coefficient *R* ^2^ = 0.984.

We observed a significant logarithmic relationship between the rate of capacitance change and the concentration of the PIK3CA-H1047R antigen within the observed concentration range of 1.0 ng/mL to 1 μg/mL. This phenomenon arises due to the saturation effect on the electrode surface, where the binding of antigen molecules to antibody sites gradually saturates available binding sites. Consequently, as the concentration of the PIK3CA-H1047R antigen increases, the rate of capacitance change slows logarithmically, eventually reaching a plateau, with further concentration increases resulting in smaller changes in capacitance. This saturation effect is commonly observed in biosensor analyses and is typically described by a logarithmic relationship between the analyte concentration and sensor response.

### 4.2 PIK3CA-H1047R antigen detection in clinical serum samples

**FIGURE 5:**
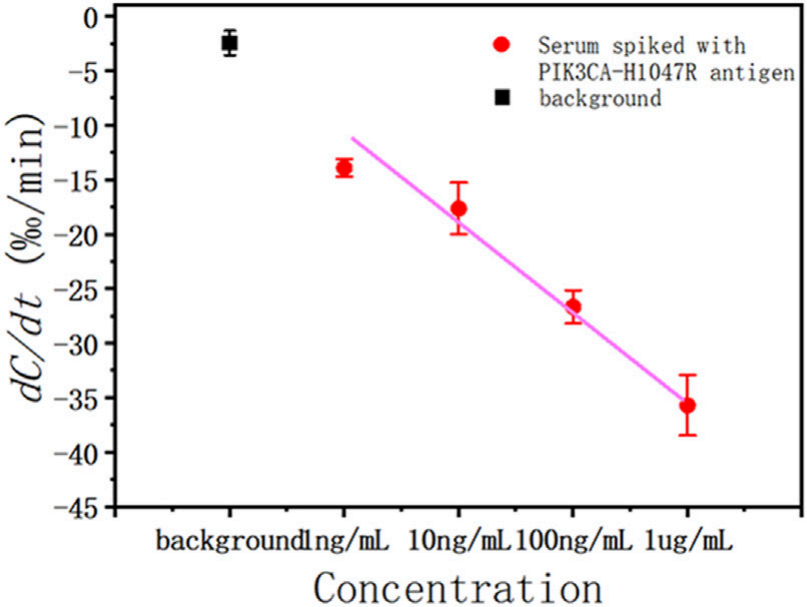
Capacitance change rate for serum spiked with various concentrations of the PIK3CA-H1047R antigen (red solid circle) and background (black solid square).

To evaluate the performance of the sensor in a complex matrix, the PIK3CA-H1047R antigen solution was added to 1:20 diluted human serum from 1 μg/mL, 100 ng/mL, 10 ng/mL, and 1 ng/mL antigen solutions. In serum samples supplemented with the PIK3CA-H1047R antigen, capacitance decreased monotonically with time due to the binding reaction. As the PIK3CA-H1047R antigen content increased, the capacitance change rate was larger, which was to be expected. The experiment was repeated five times, and Figure 5 shows the relationship between the mean response with error bars and the PIK3CA-H1047R antigen concentration.

### 4.3 Selectivity of the biosensor for PIK3CA-H1047R antigen detection in serum samples

**FIGURE 6:**
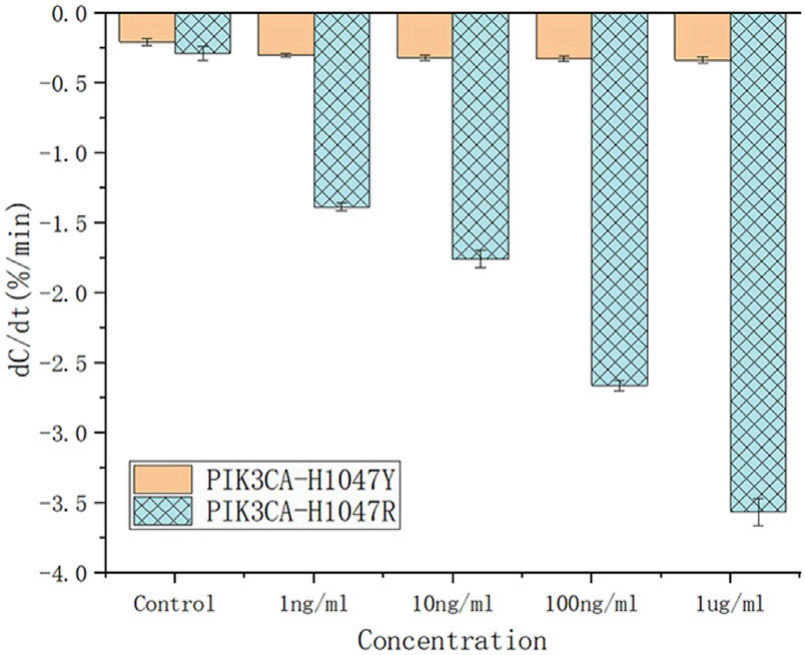
Capacitance change rate for the unspiked serum (black solid square) and serum spiked with various concentrations of PIK3CA-H1047Y antigen (brown lines) and PIK3CA-H1047R antigen (blue lines).

To evaluate the specificity of the antibody, various concentrations of the structurally similar molecule, PIK3CA-H1047Y antigen, were spiked into serum and tested for their binding affinity against the PIK3CA-H1047R antibody. PIK3CA-H1047Y antigen samples were prepared in the same way as the PIK3CA-H1047R antigen. The capacitance rate change from 1.0 ng/mL to 1.0 ug/mL was compared with those of the PIK3CA-H1047R antigen-spiked samples (Figure 6).

Based on their dC/dt values, even at 1.0 ug/mL, PIK3CA-H1047Y antigen-spiked serum samples still produced negligible responses, which is less than that of 1.0 ng/mL PIK3CA-H1047R antigen in serum samples. These results indicate there is little, if any, cross-reactivity of the PIK3CA-H1047R antibody with the PIK3CA-H1047Y antigen. All of the PIK3CA-H1047Y antigen responses fell within the cut-off values, such that the serum samples were considered to be PIK3CA-H1047R antigen-negative.

## 5 Conclusion

When a sample containing the PIK3CA-H1047R antigen was loaded on the interdigital microelectrode immobilized with the PIK3CA-H1047R antibody, the interfacial capacitance was reduced due to the binding of the antibody to the antigen. Using ACEK convection technology, we were able to detect the PIK3CA-H1047R antigen within 60 s of sample loading on the chip. The LOD of this sensor is 1 ng/mL, and the further development of this sensor provides an efficient platform for ctDNA detection. In conclusion, we developed a capacitive biosensor for rapid and label-free detection of ctDNA in biological matrices. It has the advantages of short detection time, low cost, no labeling, and high sensitivity, but it still faces the challenge of instrument miniaturization when it is promoted to practical clinical applications. Its limitations include the miniaturization of the device, the stability of the sensor performance, the reliability of signal detection, and the noise interference at small sizes. In future studies, we will explore ways to address these technical challenges to facilitate further optimization and application of biosensors. In addition, by changing the sensor-modified probe, multiple mutation types can be detected.

## Data Availability

The original contributions presented in the study are included in the article/Supplementary Material; further inquiries can be directed to the corresponding authors.
